# Global and regional estimates of COPD prevalence: Systematic review and meta–analysis

**DOI:** 10.7189/jogh.05-020415

**Published:** 2015-12

**Authors:** Davies Adeloye, Stephen Chua, Chinwei Lee, Catriona Basquill, Angeliki Papana, Evropi Theodoratou, Harish Nair, Danijela Gasevic, Devi Sridhar, Harry Campbell, Kit Yee Chan, Aziz Sheikh, Igor Rudan, Global Health Epidemiology Reference Group (GHERG)

**Affiliations:** Centre for Global Health Research and WHO Collaborating Centre for Population Health Research and Training, The Usher Institute for Population Health Sciences and Informatics, University of Edinburgh, Scotland, UK

## Abstract

**Background:**

The burden of chronic obstructive pulmonary disease (COPD) across many world regions is high. We aim to estimate COPD prevalence and number of disease cases for the years 1990 and 2010 across world regions based on the best available evidence in publicly accessible scientific databases.

**Methods:**

We conducted a systematic search of Medline, EMBASE and Global Health for original, population–based studies providing spirometry–based prevalence rates of COPD across the world from January 1990 to December 2014. Random effects meta–analysis was conducted on extracted crude prevalence rates of COPD, with overall summaries of the meta–estimates (and confidence intervals) reported separately for World Health Organization (WHO) regions, the World Bank's income categories and settings (urban and rural). We developed a meta–regression epidemiological model that we used to estimate the prevalence of COPD in people aged 30 years or more.

**Findings:**

Our search returned 37 472 publications. A total of 123 studies based on a spirometry–defined prevalence were retained for the review. From the meta–regression epidemiological model, we estimated about 227.3 million COPD cases in the year 1990 among people aged 30 years or more, corresponding to a global prevalence of 10.7% (95% confidence interval (CI) 7.3%–14.0%) in this age group. The number of COPD cases increased to 384 million in 2010, with a global prevalence of 11.7% (8.4%–15.0%). This increase of 68.9% was mainly driven by global demographic changes. Across WHO regions, the highest prevalence was estimated in the Americas (13.3% in 1990 and 15.2% in 2010), and the lowest in South East Asia (7.9% in 1990 and 9.7% in 2010). The percentage increase in COPD cases between 1990 and 2010 was the highest in the Eastern Mediterranean region (118.7%), followed by the African region (102.1%), while the European region recorded the lowest increase (22.5%). In 1990, we estimated about 120.9 million COPD cases among urban dwellers (prevalence of 13.2%) and 106.3 million cases among rural dwellers (prevalence of 8.8%). In 2010, there were more than 230 million COPD cases among urban dwellers (prevalence of 13.6%) and 153.7 million among rural dwellers (prevalence of 9.7%). The overall prevalence in men aged 30 years or more was 14.3% (95% CI 13.3%–15.3%) compared to 7.6% (95% CI 7.0%–8.2%) in women.

**Conclusions:**

Our findings suggest a high and growing prevalence of COPD, both globally and regionally. There is a paucity of studies in Africa, South East Asia and the Eastern Mediterranean region. There is a need for governments, policy makers and international organizations to consider strengthening collaborations to address COPD globally.

In a follow–up to the 2011 United Nations (UN) high level political declaration on non-communicable diseases (NCDs) [1], the World Health Assembly, in 2012, endorsed a new health goal (the “25 by 25 goal”), which focuses on reduction of premature deaths from COPD and other NCDs by 25% by the year 2025 [2]. Despite this initiative, experts have reported that COPD remains a growing [3], but neglected global epidemic [4]. The World Health Organization (WHO) estimated that there were about 62 million people with moderate to severe COPD in 2002, with the total number of COPD cases predicted to increase to about 200 million in 2010 [5,6]. According to the 2010 Global Burden of Disease (GBD) study, COPD was responsible for about 5% of global disability–adjusted life years – DALYs (76.7 million) – and 5% of total deaths (2.9 million) [7,8]. COPD is currently rated the fourth most common specific cause of death globally and predicted to be the third by 2030, in the absence of interventions that address the risks – especially tobacco smoking, exposures to combustion products of biomass fuels and environmental pollution [9,10].

The burden of COPD has been reported to be high in some high–income countries (HIC), particularly due to high prevalence of smoking in these settings [11]. For example, between years 2000 and 2010, about 4%–10% of adults were diagnosed with non–reversible and progressive airway obstruction (a basic feature of COPD) in population–based surveys across many European countries, with smoking indicated as a major risk [12]. The WHO has estimated that in many HIC up to 73% of COPD deaths are related to tobacco smoking [6]. The European Union (EU) reported that the direct cost from COPD was over 38.6 billion Euros in 2005, representing about 3% of total health care expenditure [13,14]. In the United States (US), over 2.7 million adults were estimated to have COPD in 2011, with about 135 000 deaths reported [15]. In 2010, the US government spent nearly US$ 49.9 billion on COPD, including 29.5 billion spent on direct health care, 8.0 billion on indirect morbidity and 12.4 billion on indirect mortality costs, respectively [15].

Meanwhile, it has been estimated that despite a high prevalence of COPD in some HIC, 90% of COPD deaths still occur in low– and middle–income countries (LMIC)in the future

 [4] and 40% of these deaths are attributable to smoking [6]. The burden in LMIC has been comparatively high owing to relatively low COPD awareness, challenges with COPD diagnosis and increased exposures to additional risk factors, especially combustion products of biomass fuels [16]. Salvi and colleagues reported that about 3 billion people globally are exposed to smoke from biomass fuel, compared to 1 billion people who smoke tobacco globally [17]. In many developing countries COPD is neglected by governments, physicians, experts and the pharmaceutical industry, although it's been identified as an important public health problem [4].

In the last two decades, the Burden of Obstructive Lung Disease (BOLD) initiative has been collecting country–specific data on the prevalence, risk factors and socioeconomic burden of COPD, using standardized and tested methods for conducting COPD surveys in the general population [18]. This is expected to provide governments of many nations with country–specific evidence on which to develop policy on COPD prevention and management [18]. As noted above, this initiative is yet to take a full effect in many LMIC [19]. In addition, spirometry (the gold standard for COPD diagnosis) is not widely available in many LMIC [16]. Even when it is there, professionals in LMIC are often not being trained properly on how to use spirometers or interpret spirometry results. There is concern that COPD burden has been underestimated, owing to over–reliance on doctor’s diagnosis, with many diagnoses not being based on spirometry and international diagnostic guidelines [20]. The lack of routine COPD data collation and effective health information management system in many LMIC also implies that these settings could have been grossly under–represented in global burden of COPD estimates [11].

Some global and regional estimates of COPD burden have been published [1,21–23]. However, despite the fact that COPD is now prevalent in both HIC and LMIC, experts have raised concerns that reliable estimates of COPD prevalence are still few in many parts of the world. Moreover, many of the estimates are based on varying definitions and diagnostic criteria of COPD [9]. Also, some of the current estimates were reported before the BOLD surveys in several countries, thereby failing to account for the additional spirometry–based epidemiological data from the BOLD surveys. There is a need for a revised and updated estimate of COPD prevalence across world regions. We conducted a systematic review of COPD prevalence based on spirometry data across world regions. Our aim was to provide global and regional prevalence rates of COPD that could facilitate adequate policy response in these regions.

## METHODS

### Search strategy and selection criteria

After identifying relevant Medical Subject Headings (MeSH), we conducted a systematic search of Medline, EMBASE and Global Health for studies estimating the prevalence of COPD globally from January 1990 to December 2014. We also searched Google Scholar for unpublished studies. Reference lists of retained publications were further hand-searched for studies omitted in our initial searches (see search terms in Tables s1–3 in **Online Supplementary Document(Online Supplementary Document)**).

We included original population–based (cohort or cross–sectional) studies conducted worldwide. The retained studies provided estimates of the prevalence and number of cases of COPD and/or relevant population–based information from which COPD prevalence could be estimated. We excluded studies that had unclear study designs and methodologies, conducted before 1990, on non–human subjects, and that were reviews, viewpoints or editorials. No language restrictions were applied.

### Case definitions

Based on an understanding of the diagnosis of COPD as reported by respiratory physicians, we included only studies that were based on spirometry, as these have been shown to be consistent with the diagnosis of COPD worldwide [24,25]. However, it is important to note that experts may still not fully agree on the spirometry–based definition that best defines COPD [26]. In 2001, the Global initiative on Obstructive Lung Disease (GOLD) recommended using the ratio of forced expiratory volume in one second (FEV_1_) to forced vital capacity (FVC) that is less than 70% in the diagnosis of COPD (FEV_1_/FVC<70%) [25,27]. This diagnostic criterion was also endorsed by the American Thoracic Society (ATS) and the European Respiratory Society (ERS) in 2004 [28]. It has been acknowledged that this criterion is simple and independent of reference equations [26]. However, the use of a fixed FEV_1_/FVC ratio has been debated from a number of perspectives [29–32], which we summarized in the discussion (see later). For the current study, we selected studies that used case definitions for COPD as shown in detail in **Table 1**.

**Table 1:** Basic characteristics of the studies retained for the analyses (a total of 140 study sites from 123 studies)

Characteristics	Study sites
**WHO regions:**	
AFRO	6
AMRO	15
EMRO	7
EURO	64
SEARO	6
WPRO	42
**Income category:**	
High	87
Low and middle	53
**Settings:**	
Mixed*	52
Urban	63
Rural	25
**Study period:**	
1990–1999	24
2000–2009	94
2010–present	22
**COPD case definitions:**	
FEV_1_/FVC<70%	130
FEV_1_/FVC<LLN	8
FEV_1_/FVC<75%	1
FEV_1_/FVC≤65%	1

COPD – chronic obstructive pulmonary disease, FEV – forced expiratory volume, FVC – forced vital capacity, LLN – lower limit of normal

*Studies presenting a joint COPD estimate for urban and rural settings.

### Data extraction, synthesis of results and analysis

All extracted data were stored in Microsoft Excel file format. A parallel search and extraction was conducted by three independent reviewers (SC, CL and CB). Any disagreement between the three reviewers over article inclusion, exclusion and/or data extraction for the current study was resolved through re-review of their work and agreement between their two supervisors (DA and IR). We did not calculate Kappa statistics for the agreement between all reviewers because it was not amenable to straightforward computation and interpretation in this two-stage extraction process, which was based on a mix of independent review and collaborative re-review. Also, we did not make any attempts to contact the authors of studies that were rejected based on unclear reporting. Data were abstracted systematically on sample size, mean age, number of COPD cases, and the respective age– and sex– specific prevalence rates. These were classified into WHO regions, the World Bank's income categories, and study setting (urban, rural or mixed). For studies conducted on the same study site, population or cohort, the first chronologically published study was selected, and all additional data from other studies were included in the selected paper. All extracted information from the retained studies is available in Table s4 in **Online Supplementary Document(Online Supplementary Document)**.

A random effect meta–analysis (DerSimonian and Laird method) was conducted on extracted crude prevalence rates of COPD [33]. Overall summaries of the meta–estimates (and confidence intervals) were reported separately for the WHO regions, the World Bank's income categories, and study settings, all expressed as percentages.

We developed a meta–regression epidemiological model and applied this on crude prevalence rates of COPD. In this model, mean ages corresponding to each prevalence rate were plotted on the *x*–axis (independent variable) and crude prevalence rates were plotted on the *y*–axis (dependent variable). We accounted for variation in sample sizes from each data point and controlled for year of publication to generate estimates of COPD prevalence for the years 1990 and 2000, respectively. The best fit was then used in our model and the equation generated from the fitted line was applied to the respective midpoints of the United Nations (UN) 5–year age–group population estimates for the years 1990 and 2010, respectively (United Nations, 2013). This enabled us to determine the prevalence of COPD globally and in the WHO regions. A detailed description of our model has been explained in our previous study where we estimated the burden of COPD in Africa [34]. All analyses were conducted in Stata 13.1 (Stata Corp LP, College Station, Texas, USA).

## RESULTS

### Systematic review

**Figure 1:**
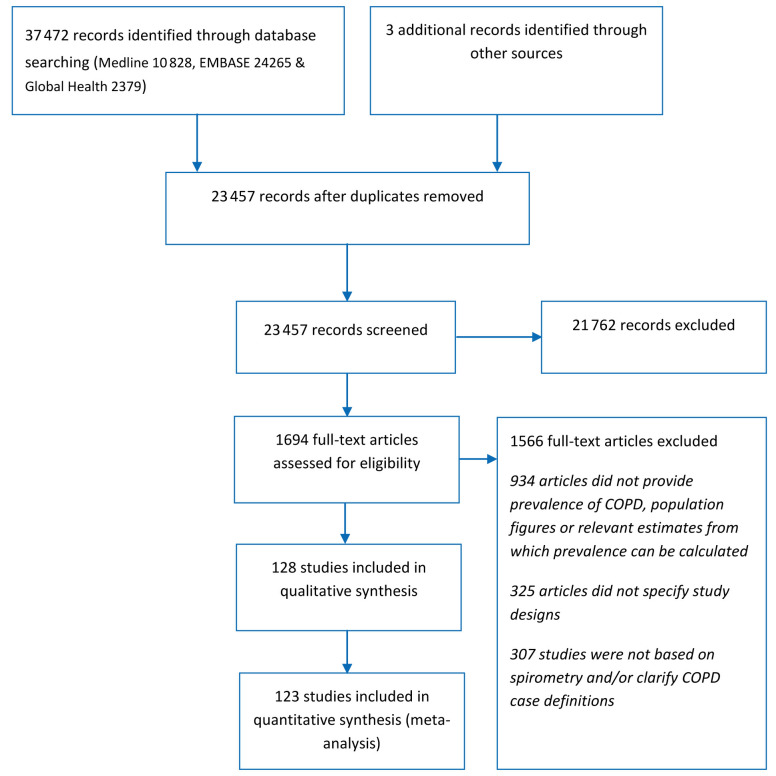
PRISMA flowchart of study selection.

Our search returned 37 472 publications: 10 828 in Medline, 24 265 in EMBASE and 2379 in Global Health. A further three studies were included from other sources (Google Scholar and hand–searching reference list of relevant publications). 23 457 studies remained after removing duplicates. After screening titles for relevance (studies estimating the burden of COPD) 21 762 studies were excluded. We therefore assessed 1694 full–text papers. After applying the quality criteria, 1566 studies were excluded. This is because 934 articles did not provide prevalence data on COPD, population figures or relevant estimates from which prevalence can be calculated; further 325 articles did not specify study designs; and 307 studies were not based on spirometry and/or clarify COPD case definitions. A total of 128 studies were retained for the review, with 123 providing quantitative data for a total of 140 study settings (see **Figure 1** for the PRISMA flow diagram of study selection).

### Study characteristics

The retained studies [23,26,35–155] were conducted across 140 locations spread across the six regions of the WHO (see Table s4 in **Online Supplementary Document(Online Supplementary Document)**). European (EURO) and Western Pacific (WPRO) regions had the highest number of studies with 64 and 42 study sites, respectively. This was followed by the American region (AMRO) with 15 study sites. Eastern Mediterranean region (EMRO) had seven study sites, while the African region (AFRO) and South East Asia region (SEARO) each had six study sites (**Table 1**). Five studies were conducted in multiple sites. They included the BOLD study [40], European Community of Respiratory Health Survey (ECRHS) [113], the PLATINO study in Latin America [23] and two other studies in Europe [61,89].

A total of 52 countries were represented, with China contributing the largest number of studies (22 in total). The mean age across studies was 54.1 years, ranging from 32 to 74 years. The total population from all studies was 877 566. The COPD survey guidelines employed across selected studies included GOLD, ATS, ATS/ERS and the British Thoracic Society (BTS) (see Table s4 in **Online Supplementary Document(Online Supplementary Document)**). However, COPD diagnosis was based on the diagnostic criterion of FEV_1_/FVC<70% in 92.2% of all retained studies (see **Table 1** and Table s4 in **Online Supplementary Document(Online Supplementary Document)** for detailed explanation of characteristics of selected studies).

### Meta–estimates from crude COPD prevalence rates

**Figure 2:**
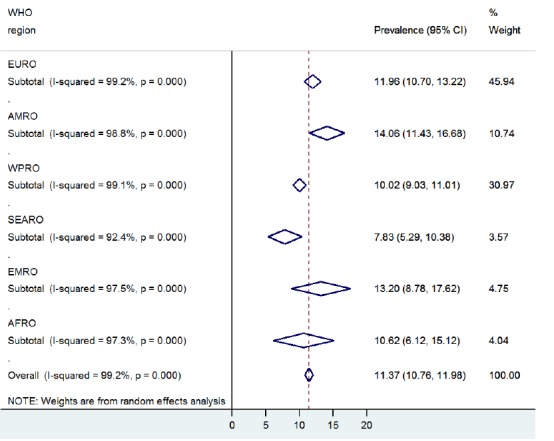
Overall pooled crude prevalence of COPD by WHO regions.

**Figure 3:**
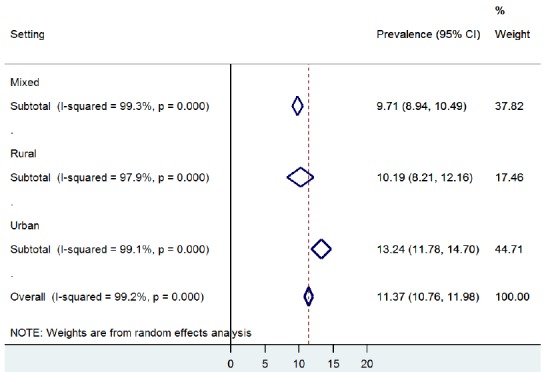
Overall pooled crude prevalence of COPD by study settings.

**Figure 4:**
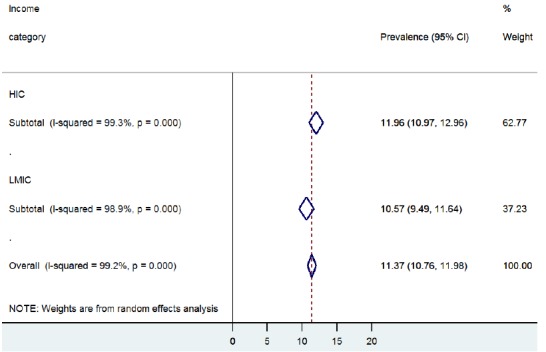
Overall pooled crude prevalence of COPD by World Bank income category.

Forest plots were used to give a visual assessment of the pooled crude prevalence along with 95% confidence intervals of COPD by WHO regions, by study settings, and by the World Bank income category (**Figures 2-4**). We used the I^2^-statistic to evaluate heterogeneity in COPD prevalence between the retained studies. From the random effects meta–analysis, a prevalence of 11.4% (CI: 10.8%–12.0%) was estimated globally in people aged ≥30 years (**Figure 2**). The overall prevalence was higher among men (14.3%; CI: 13.3%–15.3%) than women (7.6%; CI: 7.0%–8.2%) (**Table 2**).

**Table 2:** Summary of pooled crude COPD prevalence from selected studies (prevalence expressed in percentages for the population aged 30 years or older)

	Both sexes (95% CI)	Men (95% CI)	Women (95% CI)
World	11.4 (10.8–12.0)	14.3 (13.3–15.3)	7.6 (7.0–8.2)
**WHO region:**			
AFRO	10.6 (6.1–15.1)	15.1 (8.8–21.3)	8.0 (4.1–11.9)
AMRO	14.1 (11.4–16.7)	17.6 (14.6–20.6)	11.8 (9.7–13.9)
EMRO	13.2 (8.8–17.7)	15.3 (5.9–24.7)	6.0 (2.5–9.5)
EURO	12.0 (10.7–13.2)	13.7 (12.0–15.3)	7.6 (6.8–8.5)
SEARO	7.8 (5.3–10.4)	9.3 (4.7–13.9)	3.6 (2.4–4.9)
WPRO	10.0 (9.0–11.0)	14.4 (12.5–16.3)	6.4 (5.3–7.5)
**Study setting:**			
Mixed*	9.7 (8.9–10.5)	13.2 (11.8–14.6)	5.7 (5.3–6.2)
Urban	13.2 (11.8–14.7)	15.7 (13.7–17.7)	9.4 (8.3–10.6)
Rural	10.2 (8.2–12.2)	13.1 (8.5–17.7)	6.3 (4.4–8.2)
**Income category:**			
High	12.0 (11.0–13.0)	14.3 (13.0–15.6)	8.1 (7.3–8.8)
Low and middle	10.6 (9.5–11.6)	14.5 (12.3–16.7)	6.5 (5.8–7.2)

COPD – chronic obstructive pulmonary disease, CI – confidence interval

*Studies presenting a joint COPD estimate for urban and rural settings.

Among the WHO regions, AMRO region had the highest prevalence (14.1%; CI: 11.4%–16.7%), followed by EMRO (13.2%; CI: 8.8%–17.7%) and EURO (12.0%; CI: 10.7%–13.2%). AFRO had an estimated prevalence of 10.6% (CI: 6.1%–15.1%) and WPRO of 10.0% (CI: 9.0%–11.0%). SEARO had the lowest prevalence of 7.8% (CI: 5.3%–10.4%) (**Figure 2** and **Table 2**).

Across most settings, urban dwellers had higher COPD prevalence rates (13.2%; CI: 11.8%–14.7%) than rural populations (10.2%; 8.2%–12.2%) (**Figure 3** and **Table 2**). An analysis according to the World Bank's income categories revealed that the prevalence was higher in high–income countries (HIC): 12.0% (CI: 11.0%–13.0%), compared to 10.6% (CI: 9.5%–11.6%) in LMIC (**Figure 4** and **Table 2**).

### Modelled estimates of COPD prevalence and number of cases

**Figure 5:**
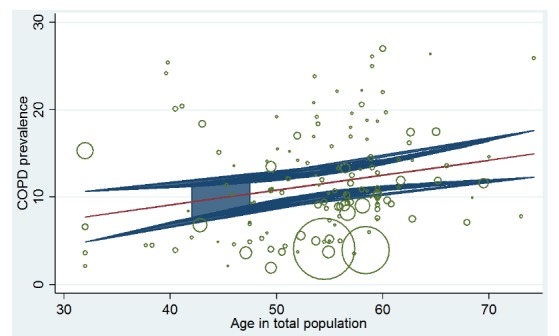
Epidemiological model based on meta–regression: The model is based on extracted crude COPD prevalence rates and adjusted for the study period and mean age of subjects from each study (the size of each bubble corresponds to the sample size from the respective study).

The initial meta–regression analysis showed a significant effect of mean age and the year of study on estimated COPD prevalence rates. We therefore controlled for these effects in the model (**Figure 5** and **Table 3**) [34]. From the model based on meta–regression we estimated about 227.3 million COPD cases in the year 1990 among people aged 30 years or more, corresponding to the global prevalence of 10.7% (7.3%–14.0%) in this age group. However, the number increased to 384 million COPD cases in 2010, corresponding to the global prevalence of 11.7% (8.4%–15.0%). This is an increase of 68.9% and it is driven mainly by the global demographic changes over the 20–year period (**Table 4**).

**Table 3:** Summary of meta–regression analysis

Prevalence	Coefficient	SE	t	*P* > t	95% CI lower	95% CI upper
Age	0.1662	0.0633	2.63	0.01	0.0410	0.2913
Year	0.0447	0.0923	0.48	0.629	–0.1377	0.2272
Constant	–87.1297	184.5720	–0.47	0.638	–452.1321	277.8727

CI – confidence interval

**Table 4:** Summary of overall COPD cases and prevalence rates in people aged 30 years or more (estimates derived from epidemiological model)

	1990		2010		% increase in COPD cases
	Cases (millions)	Prevalence (%)	Cases (millions)	Prevalence (%)	
**World**	227.3	10.7 (7.3–14.0)	384.0	11.7 (8.4–15.0)	68.9
**AFRO**	14.1	9.8 (8.9–10.7)	28.5	11.4 (10.5–12.3)	102.1
**AMRO**	41.6	13.3 (12.9–13.7)	72.0	15.2 (14.9–15.5)	73.1
**EMRO**	13.4	11.8 (10.1–13.5)	29.3	13.4 (11.8–15.1)	118.7
**EURO**	54.2	11.8 (11.6–12.0)	66.4	13.7 (13.5–13.9)	22.5
**SEARO**	44.5	7.9 (7.5–8.4)	75.1	9.7 (9.3–10.1)	68.8
**WPRO**	59.5	9.2 (9.0–9.4)	112.7	11.1 (10.9–11.3)	89.4
**Urban**	120.9	13.2 (10.0–16.4)	230.3	13.6 (11.2–16.9)	90.5
**Rural**	106.3	8.8 (6.5–11.1)	153.7	9.7 (7.6–11.8)	44.6

COPD – chronic obstructive pulmonary disease

Based on the UN percentage of people living in urban areas in 1990 (43.0%) and 2010 (51.6%) [156], we estimated the global population in these regions in those two years. Our model revealed higher prevalence and number of COPD cases among urban dwellers. In 1990, we estimated about 120.9 million COPD cases among people aged 30 years or more, accounting for a prevalence of 13.2% (10.0%–16.4%). At the same time, rural dwellers had 106.3 million cases, accounting for a prevalence of 8.8% (6.5%–11.1%). By the year 2010, the number of COPD cases among urban dwellers rose to more than 230 million, accounting for a prevalence of 13.6% (11.2%–16.9%), and about 153.7 million cases among rural dwellers, accounting for a prevalence of 9.7% (7.6%–11.8%). The percentage of increase in COPD cases between 1990 and 2010 was higher among urban dwellers than among rural residents (90.5% vs 44.6%, respectively) (**Table 5**).

**Table 5:** Estimated number of COPD cases (in thousands) and prevalence by 5–year age groups and WHO regions (estimates derived from epidemiological model)

Age (years)	AFRO		AMRO		EMRO		EURO		SEARO		WPRO		WORLD	
	**1990**	**2010**	**1990**	**2010**	**1990**	**2010**	**1990**	**2010**	**1990**	**2010**	**1990**	**2010**	**1990**	**2010**
**30–34**	2123.771	4795.619	5612.298	7809.886	2160.789	4817.729	5211.630	7524.854	8278.109	9429.498	6870.982	9683.379	30257.579	44060.964
**35–39**	2008.300	4243.869	5388.764	8020.309	1967.288	4325.147	5460.654	7595.221	7597.037	9656.235	7996.018	13297.535	30418.062	47138.315
**40–44**	1851.101	3722.464	4940.857	8193.717	1715.856	3968.634	5359.191	7509.439	5987.177	9823.378	6927.851	14807.541	26782.034	48025.172
**45–49**	1711.139	3356.237	4278.075	8705.217	1527.214	3577.205	4750.212	7363.516	5349.059	9464.399	6122.079	13200.449	23737.780	45667.023
**50–54**	1553.436	3004.563	3880.853	8293.600	1417.907	3202.697	5962.870	7009.837	4943.444	8899.492	6235.365	12475.879	23993.876	42886.069
**55–59**	1390.896	2651.316	3657.247	7479.966	1284.976	2675.335	5598.557	6476.111	4235.755	7858.034	6258.808	13161.340	22426.240	40302.102
**60–64**	1172.591	2219.778	3612.586	6401.209	1115.220	2094.671	5987.521	5532.130	3240.639	6080.409	5583.637	10297.357	20712.194	32625.554
**65–69**	923.106	1775.713	3293.108	5088.149	884.636	1658.040	5084.118	4231.163	2277.483	5002.799	4648.526	8015.963	17110.977	25771.827
**70–74**	658.987	1296.303	2640.100	4059.690	630.898	1301.601	3325.209	4808.956	1533.480	3971.306	3779.514	6852.798	12568.188	22290.654
**75–79**	397.162	827.323	2041.759	3221.004	402.706	911.945	3570.859	3588.680	739.897	2635.409	2642.263	5333.326	9794.645	16517.688
**80+**	281.660	615.016	2277.784	4770.635	307.511	760.336	3899.732	4727.433	304.694	2243.969	2403.848	5585.976	9475.228	18703.365
**Total 30+**	**14072.150**	**28508.201**	**41623.431**	**72043.382**	**13415.001**	**29293.340**	**54210.554**	**66367.341**	**44486.775**	**75064.929**	**59468.891**	**112711.542**	**227276.802**	**383988.734**
**% Prevalence 30+**	**9.79**	**11.39**	**13.31**	**15.19**	**11.82**	**13.43**	**11.83**	**13.70**	**7.92**	**9.68**	**9.20**	**11.14**	**10.67**	**11.71**
**Lower 95% CI**	8.92	10.52	12.97	14.85	10.14	11.75	11.63	13.50	7.49	9.25	9.01	10.95	7.34	8.38
**Upper 95% CI**	10.67	12.26	13.65	15.54	13.49	15.10	12.03	13.89	8.36	10.11	9.40	11.34	14.01	15.04

COPD – chronic obstructive pulmonary disease, CI – confidence interval

## DISCUSSION

This study is among the first systematic attempts to estimate the prevalence of COPD across the world regions using spirometry–based data. The estimates presented here are based on the age range starting from 30 years, while many of the previous reviews were based on people aged 40 years or older. An appreciable prevalence of COPD has been reported in younger population groups, adding to uncertainties over the current epidemiological situation globally.

In the current study, we estimated a global prevalence of 10.7% (7.3%–14.0%) in 1990 and 11.7% (8.4%–15.0%) in 2010, corresponding to 227 and 384 million of affected cases in 1990 and 2010, respectively. This estimate is an order of magnitude higher than the one presented in the 2001 World Health Report, estimating a world–wide prevalence of COPD of 10.1 per 1000 population (12.1 per 1000 men and 8.1 per 1000 women) [157]. A 2006 global review conducted by Halbert and colleagues reported a pooled prevalence of 9.2% (7.2–11.0), based on 26 spirometry–based estimates – a figure much closer to our estimates [21]. In the 2005 BOLD study, conducted in 12 sites globally and based on post–bronchodilator FEV_1_/FVC<70%, the overall prevalence of Stage II or higher COPD was 10.1% (men 11.8% and women 8.5%) [40], which is again much closer to our results. Given that the estimates presented in the 2001 World Health Report were based on doctor’s diagnosis and included all ages, this may explain the departure from spirometry-based estimates. All spirometry–based estimates to date seem to be comparable and may be indicative of the usefulness of spirometry as a gold standard for COPD diagnosis in population–based studies.

The highest COPD prevalence estimated in this study was observed in the American region, with an estimated prevalence of 13.3% and 15.2% in 1990 and 2010, respectively. Moreover, we estimated that 113 million COPD cases should be expected in Western Pacific region in 2010, almost doubling the estimated for 1990 which stood at 60 million. In the South East Asia, we estimated about 66.4 million COPD cases in 2010. Comparing our results to other estimates from previous studies, in the PLATINO multicentre study conducted across five South–American cities, which applied the same survey approach as the 2005 BOLD study, crude prevalence of COPD ranged from 7.8% in Mexico city to 19.7% in Montevideo [23]. A total of 12.2 million COPD cases, corresponding to an overall prevalence of 14.3%, was estimated for the urban population [23]. A mathematical model derived from prevalence of known COPD risk factors in 12 Asia–Pacific sites estimated about 57 million moderate–to–severe COPD cases in people aged 30 years or more in 2002, which is equivalent to a prevalence of 6.3% in the Asia Pacific region [158]. This figure is considerably larger than the WHO estimate for the region, which stood at 3.9% [157]. In addition, the regional working group reported that the number of COPD cases in China in 2002 should be expected to reach 38.2 million, with a prevalence of 6.5% [158]. This is again about 2.5 times higher than the estimates reported by the WHO [157]. Such differences in the regional pooled prevalence rates reported by separate authors may be linked to heterogeneities arising from differences in survey methodologies, population structure, subject’s age and case identification [159]. Moreover, experts have reported that despite the apparently large burden of COPD in Western Pacific and South East Asia, there are few good epidemiological surveys on COPD in these regions [22]. For example, in a recent review in India, McKay and colleagues reported that they could not identify a single high quality study that provided detailed estimate of COPD prevalence using a relatively standard spirometry-based definition, and were therefore unable to perform a meta–analysis [160]. This was also observed in our study, as there were only three studies retained in India with only two of these providing age– and sex–specific COPD prevalence rates. However, we managed to include several large epidemiological surveys conducted in China, which actually had the highest number of retained studies (22 studies) of all the 52 countries represented (see Table s4 in **Online Supplementary Document(Online Supplementary Document)**).

In the WHO EURO region, we noted the lowest increase in total number of COPD cases between 1990 and 2010 (22%). This may reflect the reduction in prevalence of smoking in Europe through intensive public health measures and legal regulations. According to the European Health Interview Survey (EHIS), the prevalence of COPD in 2008 among people aged at least 15 years in 16 EU member states ranged from 1.2% to 6.2%, with a mean prevalence of 3.1% [161], but EHIS was based on patient's self–reported diagnosis of COPD. Experts have raised concerns that COPD is often under–diagnosed when based on self-reporting of COPD diagnosis, even in high–income settings, and that the true prevalence rates may be considerably higher [162].

Several authors have reported that the epidemiological evidence on COPD in African region is still very limited and that this has affected the response to its growing burden in the region [16,34,163,164]. We estimated a prevalence of about 11% in 2010, amounting to 29 million COPD cases in the region. This is similar to a recent continent–wide estimate of more than 26 million cases in 2010 [34]. Epidemiological data are also very limited in the Middle East and North Africa [165]. Based on scarce available evidence, it appears that the EMR had the lowest absolute number of COPD cases in 1990 among the world regions, with 13 million (prevalence of 12%). However, the region then recorded the highest percentage increase by the year 2010 of 119%. Smoking rates in the Middle East and in many Mediterranean countries have reportedly been high [165]. This may imply that the burden of COPD in EMRO region could have been considerably under–estimated in the year 1990.

Several studies reported that urbanization is an important risk in the development of COPD [9,39]. We estimated over 230 million cases of COPD among global urban dwellers in 2010, accounting for almost 60% of all COPD cases. The rapid rate of urbanization in many parts of the world, especially in LMIC, may contribute to an increasing prevalence of COPD globally [39]. Moreover, the prevalence of COPD among men was consistently higher than in women across all world regions, settings and income categories. Some authors question the independent etiological role of gender in the development of COPD [166], given that the risk profile among men favours the development of disease. Recent reviews suggested that increased tobacco use among women in high–income countries and the higher risk of exposure to indoor air pollution (such as biomass fuels used for cooking and heating) in low– and middle income countries may contribute to reducing gender differences in COPD in future [1].

Our study has a number of limitations. Although we identified a considerable number of studies for this review, they were not proportionately distributed across the WHO regions, nor were the sample sizes from the regions proportional to regional populations. More than 46% of the data points were from the European region, meaning that the overall results may over–represent the burden of COPD in Europe. On the other hand, African region (4%), South East Asian region (4%) and Eastern Mediterranean region (5%) are all grossly under–represented, highlighting the lack of good quality prevalence data outside of the high–income countries.

Another important limitation relates to the differences in case definitions and diagnostic guidelines employed across studies over time. In our analysis, we required spirometry as the standard diagnostic parameter, given the concerns raised over alternative definitions of COPD. Across all retained studies, the definition based on FEV/FVC<70% was used in the large majority of studies, but this still does not address all possible sources of variation in case definition. Some authors even question the use of a fixed FEV_1_/FVC ratio mainly because it has no statistical basis and because choosing 0.7 as a cut, off point – instead of 0.68 or 0.72, for example – is essentially arbitrary [29]. Experts have argued that this fixed criterion may potentially over–diagnose COPD in the elderly, as lung elasticity decreases with age, which reduces FEV_1_ more than FVC [30]. Hence, using a fixed ratio can result in under–diagnoses in younger patients, and more frequent diagnoses in the elderly. The use of a lower limit of normal (LLN) has been suggested instead, and this is described as the lower fifth percentile of a reference population [31]. It is calculated by subtracting the standard deviation (multiplied by 1.64) from the mean [31]. However, some studies suggest that LLN may miss subjects with COPD [32]. This variation in COPD diagnosis is especially pertinent in LMICs, where undiagnosed or poorly treated asthma, bronchiectasis, tuberculosis or some other obstructive airway disease may be more prevalent and possibly misdiagnosed as COPD [17]. Even when the FEV/FVC<70% criterion was consistently applied, some studies were based on pre–bronchodilator values, rather than post–bronchodilator values, as recommended by GOLD. In addition, it was often not fully explained how exactly was spirometry performed and what was the protocol. A number of technical issues could have affected the estimates, such as the choice of spirometer, the level of training of the operator, and the process of collection and storage of spirometry measurements. Finally, physicians’ knowledge of guidelines can also pose a barrier to spirometry use in settings where it is available [167].

An additional limitation is associated with the choice of our epidemiological model, which was mainly based on age of the examinees. There are several other important predictors that could have been incorporated into the model if the information was available from the retained studies, including those related to study sites, income levels, smoking, socio–economic determinants of health, occupational exposures and others. However, the studies that we retained would rarely report these important covariates, so we could not use them in our model.

Meanwhile, it is worth noting that most of the studies (83%) included in this review were published after the year 2000, and encouragingly, the number of epidemiological studies focusing on COPD has been steadily increasing over the past two decades. This upward trend provides an indication of an increased awareness and recognition of COPD as a growing global health burden, and the need to strengthen research base and improve and standardize the methods.

Our findings suggest a high and growing prevalence of COPD, both globally and regionally, with substantial variation in trends between different world regions. The estimates presented here are consistent with other spirometry–based reviews on the burden of COPD. As with any other public health problem, increased political commitment and funding remains crucial, particularly in LMIC settings. Governments and policymakers must consider strengthening regulations to address occupational and environmental risk factors, regulate tobacco use and improve public awareness. A combined use of patient- and physician targeted educational interventions could also help [168]. The efforts of BOLD, aiming to standardize methodology and definitions, must be supported, and other research entities should strongly consider adopting similar methods or collaborating with BOLD in order to provide epidemiological results that are more comparable in and among populations. It is only through such concerted effort that the current high global COPD burden may be reduced in the coming decades.
