# Supplementary material for: Global and regional estimates of COPD prevalence: Systematic review and meta–analysis
Source: J Glob Health. 2015 Dec 20;5(2):020415. doi: 10.7189/jogh.05-020415 (PMC4693508; doi:10.7189/jogh.05-020415)
Supplement: Online Supplementary Document [file jogh-05-020415-s001.pdf]

Online Supplementary Document

Adeloye et al. Global and regional estimates of COPD prevalence: Systematic review and meta-analysis

J Glob Health 2015;5:020415

Supplementary Table 1. Search terms (MEDLINE)

#	Searches
1	exp vital statistics/ or exp incidence/
2	(incidence* or prevalence* or morbidity or mortality).tw.
3	(disease adj3 burden).tw.
4	exp "cost of illness"/
5	exp quality-adjusted life years/
6	QALY.tw.
7	Disability adjusted life years.mp.
8	(initial adj2 burden).tw.
9	exp risk factors/
10	1 or 2 or 3 or 4 or 5 or 6 or 7 or 8 or 9
11	exp Emphysema/
12	copd.mp.
13	exp bronchitis, chronic/ or exp pulmonary disease, chronic obstructive/ or exp pulmonary emphysema/
14	chronic obstructive airway disease.mp. [mp=title, abstract, original title, name of substance word, subject heading word, keyword heading word, protocol supplementary concept word, rare disease supplementary concept word, unique identifier]
15	coad.mp. [mp=title, abstract, original title, name of substance word, subject heading word, keyword heading word, protocol supplementary concept word, rare disease supplementary concept word, unique identifier]
16	11 or 12 or 13 or 14 or 15
17	10 and 16
18	limit 17 to yr="1990 -Current"

Supplementary Table 2. Search Terms (EMBASE)

#	Searches
1	exp vital statistics/ or exp incidence/
2	(incidence* or prevalence* or morbidity or mortality).tw.
3	(disease adj3 burden).tw.
4	exp “cost of illness”/
5	exp quality-adjusted life years/
6	QALY.tw.
7	Disability adjusted life years.mp.
8	(initial adj2 burden).tw.
9	exp risk factors/
10	1 or 2 or 3 or 4 or 5 or 6 or 7 or 8 or 9
11	exp Emphysema/
12	copd.mp.
13	coad.mp. [mp=abstract, title, original title, broad terms, heading words]
14	chronic bronchitis.mp. [mp=abstract, title, original title, broad terms, heading words]
15	exp chronic obstructive pulmonary disease/
16	11 or 12 or 13 or 14 or 15
17	10 and 16
18	**limit 21 to yr=”1990 -Current”**

Supplementary Table 3. Search Terms (GLOBAL HEALTH)

#	Searches
1	exp vital statistics/ or exp incidence/
2	(incidence* or prevalence* or morbidity or mortality).tw.
3	(disease adj3 burden).tw.
4	exp “cost of illness”/
5	exp quality-adjusted life years/
6	QALY.tw.
7	Disability adjusted life years.mp.
8	(initial adj2 burden).tw.
9	exp risk factors/
10	1 or 2 or 3 or 4 or 5 or 6 or 7 or 8 or 9
11	exp Emphysema/
12	copd.mp.
13	coad.mp. [mp=abstract, title, original title, broad terms, heading words]
14	chronic bronchitis.mp. [mp=abstract, title, original title, broad terms, heading words]
15	exp chronic obstructive pulmonary disease/
16	11 or 12 or 13 or 14 or 15
17	10 and 16
18	**limit 17 to yr="1990 -Current"**

Supplementary Table 4. Characteristics of retained COPD studies globally

Study	Study period	Country	Income category	WHO region	Setting	Diagnosis criteria	Mean age	Sample size	COPD cases	Prevalence
**Khelafi et al**	2010	Algeria	LMIC	AFRO	Mixed	GOLD	47.5	1800	87	4.9
**Martins et al**	2006	Cape Verde	LMIC	AFRO	Mixed	GOLD	41.7	274	23	8.4
**Fullerton et al**	2001	Malawi	LMIC	AFRO	Mixed	GOLD	46	332	45	13.6
**Gathuru et al**	1999	Nigeria	LMIC	AFRO	Urban	LLN	47.6	270	25	9.3
**Musafiri et al**	2009	Rwanda	LMIC	AFRO	Mixed	ATS/ERS	38.3	1824	82	4.5
**Buist et al**	2005	South Africa	LMIC	AFRO	Urban	GOLD	53.6	847	202	23.8
**Tan et al**	2007	Canada	HIC	AMRO	Urban	GOLD/LLN	57	2916	487	11.6
**Al-Hazmi et al**	1994	Canada	HIC	AMRO	Urban	GOLD	32	2819	186	6.6
**Tilert et al**	2009	USA	HIC	AMRO	Urban	GOLD/LLN	59.5	5447	763	10.2
**Methvin et al**	2005	USA	HIC	AMRO	Urban	GOLD	57.1	508	100	19.6
**Mannino et al**	1991	USA	HIC	AMRO	Urban	GOLD	42.8	16084	1367	6.8
**Ford et al**	2010	USA	HIC	AMRO	Urban	GOLD	49.5	9024	1218	13.5
**Vaz Fragoso et al**	2000	USA	HIC	AMRO	Urban	GOLD	60	3502	947	27.0
**Menezes et al**	2001	Brazil	LMIC	AMRO	Urban	GOLD	51.2	191	29	15.2
**Caballero et al**	2004	Colombia	LMIC	AMRO	Urban	GOLD	55.8	5539	494	8.9
**Laniado-Laborin et al**	2008	Mexico	LMIC	AMRO	Urban	GOLD	58	2293	472	20.6
**Menezes et al**	2003	Brazil	LMIC	AMRO	Urban	GOLD	55.2	1000	158	15.8
**Menezes et al**	2003	Chile	HIC	AMRO	Urban	GOLD	57	1208	204	16.9
**Menezes et al**	2003	Mexico	LMIC	AMRO	Urban	GOLD	55.9	1063	83	7.8
**Menezes et al**	2003	Uruguay	HIC	AMRO	Urban	GOLD	60.3	943	186	19.7
**Menezes et al**	2003	Venezuela	LMIC	AMRO	Urban	GOLD	51.1	1357	164	12.1
**Amra et al**	2010	Iran	LMIC	EMRO	Mixed	ERS	53.5	420	71	16.9
**Golshan et al**	1998	Iran	LMIC	EMRO	Mixed	GOLD	51.95	4636	788	17.0
**Golshan et al**	2010	Iran	LMIC	EMRO	Mixed	GOLD	58	279	62	22.2
**Waked et al**	2010	Lebanon	LMIC	EMRO	Mixed	GOLD/LLN	57.1	2201	284	12.5
**Daldoul et al**	2012	Tunisia	LMIC	EMRO	Urban	GOLD	59.5	661	49	7.4
**Al Zaabi et al**	2010	UAE	HIC	EMRO	Urban	GOLD	52	520	19	3.7
**Al Ghobain**	2011	Saudi Arabia	HIC	EMRO	Urban	GOLD	52	501	71	14.2
**Stav et al**	2007	Israel	HIC	EURO	Mixed	GOLD	60	1058	233	22.0
**Weiss et al**	2007	Austria	HIC	EURO	Mixed	GOLD	58.5	775	58	16.6
**Schirnhofer et al**	2005	Austria	HIC	EURO	Urban	GOLD	59	1258	328	26.1
**Maio et al**	2009	Austria	HIC	EURO	Urban	GOLD	41.1	1526	311	20.4
**Fabricius et al**	2003	Denmark	HIC	EURO	Urban	GOLD	62.6	4908	853	17.4
**Hansen et al**	2005	Denmark	HIC	EURO	Mixed	GOLD	65.2	4535	541	11.9
**Maio et al**	2009	Denmark	HIC	EURO	Urban	GOLD	39.8	1037	263	25.4
**Kainu et al**	2002	Finland	HIC	EURO	Urban	GOLD/LLN	49.1	628	37	5.9
**Kotaniemi et al**	2004	Finland	HIC	EURO	Rural	GOLD	47.9	683	64	9.4
**Isoaho et al**	1991	Finland	HIC	EURO	Rural	FEV1/FVC≤65%	73	1196	82	7.8
**Kanervisto et al**	2001	Finland	HIC	EURO	Mixed	GOLD	55	6525	338	5.2
**Roche et al**	2007	France	HIC	EURO	Urban	GOLD	62.8	4335	325	7.5
**Geldmacher et al**	2005	Germany	HIC	EURO	Mixed	GOLD	57.9	683	90	13.2

**Gingter et al**	2007	Germany	HIC	EURO	Urban	GOLD	59.5	432	40	9.3
**Schikowski et al***	1994	Germany	HIC	EURO	Urban	GOLD	.	.	.	.
**Maio et al**	2009	Germany	HIC	EURO	Urban	GOLD	43	4576	841	18.4
**Sichletidis et al**	2001	Greece	HIC	EURO	Mixed	GOLD	52.3	6112	341	5.6
**Minas et al**	2008	Greece	HIC	EURO	Rural	GOLD	53.9	1526	281	18.4
**Benediktsdottir et al**	2004	Iceland	HIC	EURO	Urban	GOLD	57	755	136	18.0
**Viegi et al**	1990	Italy	HIC	EURO	Rural	GOLD	44.6	1727	261	15.1
**Vanfleteren et al**	2008	Netherlands	HIC	EURO	Urban	GOLD/LLN	59.5	592	140	18.7
**Van Durme et al**	2003	Netherlands	HIC	EURO	Urban	GOLD	69.5	7983	928	11.6
**Afonso et al**	2007	Netherlands	HIC	EURO	Mixed	GOLD	58.4	185325	7308	3.9
**Waatevik et al**	2004	Norway	HIC	EURO	Mixed	GOLD	54.7	1664	228	8.7
**Hvidsten et al**	1999	Norway	HIC	EURO	Mixed	GOLD	60.8	3305	303	9.2
**Buist et al**	2005	Norway	HIC	EURO	Urban	GOLD	58.5	658	124	18.8
**Bakke et al**	1990	Norway	HIC	EURO	Mixed	GOLD	42	1275	65	5.4
**Johannessen et al**	2003	Norway	HIC	EURO	Mixed	GOLD	46	869	40	4.6
**Bednarek et al**	2007	Poland	HIC	EURO	Mixed	LLN	56.7	1960	183	9.3
**Nizankowska-Mogilnicka et al**	2005	Poland	HIC	EURO	Mixed	GOLD	55.7	526	116	22.1
**Plywaczewski et al**	2001	Poland	HIC	EURO	Urban	GOLD	49.4	676	72	10.7
**Paprzycki et al**	2000	Poland	HIC	EURO	Rural	GOLD	49.6	643	73	11.0
**Siatkowska et al**	2009	Poland	HIC	EURO	Mixed	GOLD	58.7	1026	62	6.0
**Barbara et al**	2008	Portugal	HIC	EURO	Urban	GOLD	62.8	710	101	14.2
**Cardoso et al**	1995	Portugal	HIC	EURO	Mixed	GOLD	56.4	9061	815	9.0
**Cardoso et al**	2002	Portugal	HIC	EURO	Mixed	GOLD	48.6	1384	73	5.3
**Mascarenhas et al**	2001	Portugal	HIC	EURO	Urban	GOLD	58.5	758	81	10.7
**Grzetic-Romcevic et al**	2005	Slovenia	HIC	EURO	Urban	GOLD	56.5	770	79	10.3
**Miravitlles et al**	2007	Spain	HIC	EURO	Mixed	GOLD	56.6	3802	386	10.2
**Peña et al**	1997	Spain	HIC	EURO	Mixed	ERS	53.9	3981	363	9.1
**Cabrera-Lopez et al**	2012	Spain	HIC	EURO	Urban	GOLD	55	1001	73	7.3
**Danielsson et al**	2007	Sweden	HIC	EURO	Urban	LLN	58.9	548	86	10.0
**Lindberg et al**	1996	Sweden	HIC	EURO	Rural	GOLD	61.5	1237	177	14.3
**Lindberg et al**	1994	Sweden	HIC	EURO	Rural	GOLD	49.1	666	94	14.1
**Lindberg et al**	1996	Sweden	HIC	EURO	Rural	GOLD	61.5	963	107	11.1
**Ekberg-Aronsson et al**	2002	Sweden	HIC	EURO	Mixed	GOLD	32	22044	3364	15.3
**Lindström et al**	1994	Sweden	HIC	EURO	Rural	BTS	50.5	1580	166	10.5
**Hasselgren et al**	1995	Sweden	HIC	EURO	Rural	BTS	45.4	206	4	2.1
**Larsson et al**	1991	Sweden	HIC	EURO	Mixed	ATS	49.5	1970	87	10.8
**Maio et al**	2009	Sweden	HIC	EURO	Urban	GOLD	40.5	2417	486	20.1
**Bridevaux et al**	2002	Switzerland	HIC	EURO	Mixed	GOLD	53.7	6126	307	5.0
**Arslan et al**	2012	Turkey	LMIC	EURO	Urban	GOLD	55.8	946	126	13.3
**Erdogan et al**	2012	Turkey	LMIC	EURO	Rural	GOLD	55	500	25	5.0

**Deveci et al**	2007	Turkey	LMIC	EURO	Mixed	GOLD	37.7	1188	53	4.5
**Gunen et al**	2007	Turkey	LMIC	EURO	Mixed	ATS/ERS	47.5	1160	80	6.9
**Buist et al**	2005	Turkey	LMIC	EURO	Mixed	GOLD	53.8	806	155	19.2
**Jordan et al**	1998	UK	HIC	EURO	Mixed	GOLD	58.1	18817	1684	9.0
**Melville et al**	2003	UK	HIC	EURO	Urban	GOLD	59	845	211	25.0
**Murtagh et al**	2000	UK	HIC	EURO	Mixed	BTS	55.5	722	49	6.8
**Dickinson et al**	1998	UK	HIC	EURO	Mixed	GOLD	68.4	353	35	9.9
**Renwick et al**	1993	UK	HIC	EURO	Mixed	GOLD	64.5	246	65	26.4
**Shahab et al**	2001	UK	HIC	EURO	Mixed	GOLD	56.5	8215	1093	13.3
**Maio et al**	2009	UK	HIC	EURO	Urban	GOLD	39.6	825	200	24.2
**de Marco et al**	1992	International	HIC	EURO	Mixed	GOLD	32	1942	70	3.6
**Zachariades et al**	2012	Cyprus	HIC	EURO	Mixed	GOLD	54.5	1233	60	4.9
**Islam et al**	2012	Bangladesh	LMIC	SEARO	Urban	GOLD	45.3	900	103	11.4
**Mosharraf-Hossain et al**	2009	Bangladesh	LMIC	SEARO	Urban	GOLD	53.7	400	50	12.5
**Maranetra et al**	1998	Thailand	LMIC	SEARO	Urban	GOLD	67.9	3094	220	7.1
**Parasuramalu et al**	2013	India	LMIC	SEARO	Rural	GOLD	50.8	1400	61	4.4
**Johnson et al***	2007	India	LMIC	SEARO	Rural	GOLD		.	.	.
**Mahesh et al**	2009	India	LMIC	SEARO	Rural	GOLD	44.5	350	17	4.9
**Toelle et al**	2008	Australia	HIC	WPRO	Mixed	GOLD	58.9	3357	487	14.5
**Matheson et al**	2004	Australia	HIC	WPRO	Urban	GOLD	57.3	1213	42	3.5
**Shirtcliffe et al**	2004	New Zealand	HIC	WPRO	Urban	GOLD	50.1	749	116	15.5
**Buist et al**	2005	China	LMIC	WPRO	Urban	GOLD	54	473	54	11.4
**Liu et al**	2003	China	LMIC	WPRO	Mixed	GOLD	57	3286	310	9.4
**Zhong et al**	2003	China	LMIC	WPRO	Mixed	GOLD	56.7	20245	1668	8.2
**Qiu et al**	2012	China	LMIC	WPRO	Mixed	GOLD	56	4055	360	8.9
**Rong et al**	2009	China	LMIC	WPRO	Rural	GOLD	40.5	2568	100	3.9
**Li et al**	2008	China	LMIC	WPRO	Urban	GOLD	59.5	1518	194	12.8
**Gong et al**	2009	China	LMIC	WPRO	Urban	GOLD	70	710	104	14.6
**Ling et al**	2010	China	LMIC	WPRO	Rural	GOLD	49.5	3489	138	4.0
**Ma et al**	2012	China	LMIC	WPRO	Rural	GOLD	59.5	1554	173	11.1
**Zeng et al**	2009	China	LMIC	WPRO	Urban	GOLD	60.4	3687	354	9.6
**Li et al**	2011	China	LMIC	WPRO	Rural	GOLD	59.5	1384	136	9.8
**Liu et al**	2009	China	LMIC	WPRO	Rural	GOLD	65	5420	947	17.5
**Liu et al**	2007	China	LMIC	WPRO	Mixed	GOLD	54.5	3286	310	9.4
**Zhang et al**	2008	China	LMIC	WPRO	Rural	GOLD	59.5	2001	172	8.6
**Yu et al**	2008	China	LMIC	WPRO	Rural	GOLD	55.5	1948	209	10.7
**Wang et al**	2010	China	LMIC	WPRO	Urban	GOLD	59.5	2055	156	7.6
**Jiang et al**	2006	China	LMIC	WPRO	Rural	GOLD	59.5	1883	186	9.9
**Yao et al**	2004	China	LMIC	WPRO	Rural	GOLD	59.5	1624	148	9.1
**Wang et al**	2004	China	LMIC	WPRO	Rural	GOLD	54.3	1468	176	12.0
**Yin et al**	2006	China	LMIC	WPRO	Urban	GOLD	61.7	6497	773	11.9
**Smith et al**	2008	China	LMIC	WPRO	Mixed	GOLD	54.5	317399	13013	4.1
**Zhou et al**	2009	China	LMIC	WPRO	Mixed	GOLD	54.9	12471	462	3.7

**Ko et al**	2006	Hong Kong	HIC	WPRO	Urban	GOLD	74.2	1008	261	25.9
**Lau et al**	2003	Hong Kong	HIC	WPRO	Urban	GOLD	50	525	101	19.2
**Nishimura et al**	2011	Japan	HIC	WPRO	Urban	GOLD	56	1333	67	5.0
**Fukahori et al**	2005	Japan	HIC	WPRO	Urban	GOLD	66	1424	193	13.6
**Takemura et al**	1999	Japan	HIC	WPRO	Urban	GOLD	47.1	12760	459	3.6
**Fukuchi et al**	2000	Japan	HIC	WPRO	Mixed	GOLD	58	2343	256	10.9
**Kojima et al**	2002	Japan	HIC	WPRO	Urban	GOLD	49.5	11460	213	1.9
**Yoo et al**	2008	South Korea	HIC	WPRO	Mixed	GOLD	59	2501	335	13.4
**Hwang et al**	2008	South Korea	HIC	WPRO	Mixed	GOLD	59.5	6934	894	10.3
**Kim et al**	2003	South Korea	HIC	WPRO	Urban	GOLD	50.5	3642	134	3.7
**Kim et al**	2002	South Korea	HIC	WPRO	Mixed	GOLD	62.5	1673	288	16.2
**Shin et al**	2000	South Korea	HIC	WPRO	Urban	ATS	45.8	1160	119	10.3
**Fishwick et al**	1996	New Zealand	HIC	WPRO	Mixed	FEV1/FVC<75 %	32	1132	24	2.1
**Idolor et al**	2010	Philippines	LMIC	WPRO	Rural	GOLD	53.5	722	141	20.8
**Buist et al**	2005	Philippines	LMIC	WPRO	Urban	GOLD	52.9	893	124	13.9
**Lam et al**	2013	Philippines	LMIC	WPRO	Mixed	GOLD	47.5	565	40	7.1

**no overall estimate reported*
